# Microneedles loaded with cerium-manganese oxide nanoparticles for targeting macrophages in the treatment of rheumatoid arthritis

**DOI:** 10.1186/s12951-024-02374-y

**Published:** 2024-03-11

**Authors:** Tian Xia, Yuting Zhu, Kaiqiang Li, Ke Hao, Yingqian Chai, Hongyi Jiang, Chao Lou, Jiachen Yu, Wei Yang, Jilong Wang, Junjie Deng, Zhen Wang

**Affiliations:** 1 Laboratory Medicine Center, Allergy Center, Department of Transfusion Medicine, Zhejiang Provincial People’s Hospital ，Affiliated People’s Hospital, Hangzhou Medical College, Hangzhou, Zhejiang 310014 China; 2 Joint Centre of Translational Medicine, The First Affiliated Hospital of Wenzhou Medical University, Wenzhou, 325000 Zhejiang China; 3 Joint Centre of Translational Medicine, Wenzhou Institute, University of Chinese Academy of Sciences, Wenzhou, 325000 Zhejiang China; 4 Laboratory Medicine Center, Department of Transfusion Medicine, Tiantai People’s Hospital of Zhejiang Province (Tiantai Branch of Zhejiang Provincial People’s Hospital), Hangzhou Medical College, Taizhou, 317200 Zhejiang China; 5 Zhejiang Engineering Research Center for Tissue Repair Materials, Wenzhou Institute, University of Chinese Academy of Sciences, Wenzhou, 325000 Zhejiang China; 6 Department of Biophysics, Department of Neurology of the Fourth Affiliated Hospital, Zhejiang University School of Medicine, Hangzhou, 310000 China

**Keywords:** Cerium-manganese oxide nanoparticles, Microneedles, Anti-inflammatory, Rheumatoid arthritis

## Abstract

**Background:**

Rheumatoid arthritis (RA) is a prevalent inflammatory autoimmune disease characterised by persistent inflammation and joint damage with elevated levels of reactive oxygen species (ROS). Current treatment modalities for RA have significant limitations, including poor bioavailability, severe side effects, and inadequate targeting of inflamed joints. Herein, we synthesised cerium/manganese oxide nanoparticles (NPs) as efficient drug carriers with antioxidant and catalytic-like functions that can eliminate ROS to facilitate the polarization of macrophages phenotype from M1 to M2 and alleviate inflammation. Methotrexate (MTX), a first-line RA medication, was loaded into the NPs, which were further modified with bovine serum albumin (BSA) and integrated into dissolving hyaluronic acid-based microneedles (MNs) for transdermal delivery.

**Result:**

This innovative approach significantly enhanced drug delivery efficiency, reduced RA inflammation, and successfully modulated macrophage polarization toward an anti-inflammatory phenotype.

**Conclusion:**

This research not only presents a promising drug delivery strategy for RA but also contributes broadly to the field of immune disease treatment by offering an advanced approach for macrophage phenotypic reprogramming.

**Supplementary Information:**

The online version contains supplementary material available at 10.1186/s12951-024-02374-y.

## Introduction

Rheumatoid arthritis (RA) is an autoimmune disorder characterised by persistent synovitis, compromised joint function, joint inflammation, synovial proliferation, pannus development, and subsequent degradation of bone and cartilage [1, 2]. RA is often associated with chronic joint pain, oedema, and rigidity and can culminate in significant cardiovascular, pulmonary, psychological, and osseous complications [3, 4]. Globally, RA affects roughly 0.5% of the adult population, with females exhibiting a 2–3 fold higher incidence compared to males [5]. Substantial researches have revealed that the mechanism of RA is related to the increased concentrations of reactive oxygen species (ROS) [6]. Elevated levels of ROS (including superoxide anions, hydroxyl radicals, hydrogen peroxide, and singlet oxygen) induce oxidative stress and hypoxia, not only leading to lipid peroxidation, protein oxidation, and augmented DNA damage but also activating transcription factors, such as hypoxia-inducible factor-1 (HIF-1α) and vascular endothelial growth factor (VEGF) [7]. VEGF initiates pro-inflammatory gene expression and induces pro-inflammatory macrophage phenotype (M1) via the toll-like receptors (TLR) /nuclear factor kappa-B (NF-kB) pathway [8, 9]. During the initial phases of inflammation, M1 macrophages instigate inflammatory reactions through the generation of effector molecules, including ROS, nitric oxide, pro-inflammatory cytokines (interleukin (IL)-1β, tumor necrosis factor (TNF)-α), and chemokines. This, in turn, augments the activation of chondrocytes and synovial fibroblasts, ultimately leading to cartilage abnormalities and synovial hyperplasia [7, 9–11]. Conversely, M2 macrophages release anti-inflammatory cytokines associated with tissue reconstruction and immunoregulatory processes, facilitating the alleviation of inflammation and improving the pathogenesis of RA. Thus, scavenging excess ROS and maintaining the M1/M2 balance might be a promising strategy for effective treatment and promoting immune homeostasis in RA [7, 9–11].

Currently, there is no cure for RA with existing medical treatments, and primary treatment drugs include disease-modifying anti-rheumatic drugs (DMARDs), nonsteroidal anti-inflammatory drugs (NSAIDs), glucocorticoids, and biological DMARDs [12, 13]. MTX has historically served as the cornerstone medication and benchmark for RA treatment [14], but it has first-pass metabolism, poor bioavailability, and severe side effects, such as bone marrow suppression and gastrointestinal inflammation. Long-term and frequent injections of MTX can reduce patient compliance and even induce infections [15, 16]. Nanomedicine, as an emerging therapeutic approach, involves the loading of drugs onto nanoparticles to enhance drug targeting and sustain release [17]. During the progression of RA, there is a significant increase in vascular permeability attributed to angiogenesis and infiltration of inflammatory cells. By harnessing the “extravasation through leaky vasculature and subsequent inflammatory cell-mediated sequestration (ELVIS)” mechanism [18], nanomedicines exhibit a preferential localization at sites of inflammation [19–22], thereby facilitating optimized drug deposition and mitigating potential adverse reactions [23, 24]. Additionally, macrophages with M1 polarization exhibit elevated expression levels of specific receptors on their cellular membrane, including but not limited to folate receptors, scavenger receptors, and CD44. This observation paves the way for the conceptualization and development of precision-targeted delivery platforms via ligand modification on the nanoparticle exteriors [25].

However, upon entering the bloodstream, NPs are prone to rapid clearance by cells in the mononuclear phagocyte system (MPS) [26], especially macrophages, which greatly reduces the expected therapeutic effect of the disease. In order to efficiently deliver NPs for RA treatment, a transdermal delivery system provides an ideal alternative route of administration. Microneedles (MNs), as an emerging percutaneous drug delivery system, consist of arrays of micro-sized needle tips attached to a base. The needles, typically 10-2000 μm long and 10–50 μm wide, can penetrate the stratum corneum to produce microchannels and deliver drugs in a minimally invasive way [27–29]. In addition to circumventing first-pass effects, MNs enable sustained drug release, mitigate side effects, and allow for self-administration, thereby significantly enhancing patient compliance [30, 31]. Therefore, MNs are promising drug delivery systems for improving the therapeutic efficacy of RA treatment.

**Scheme 1:**
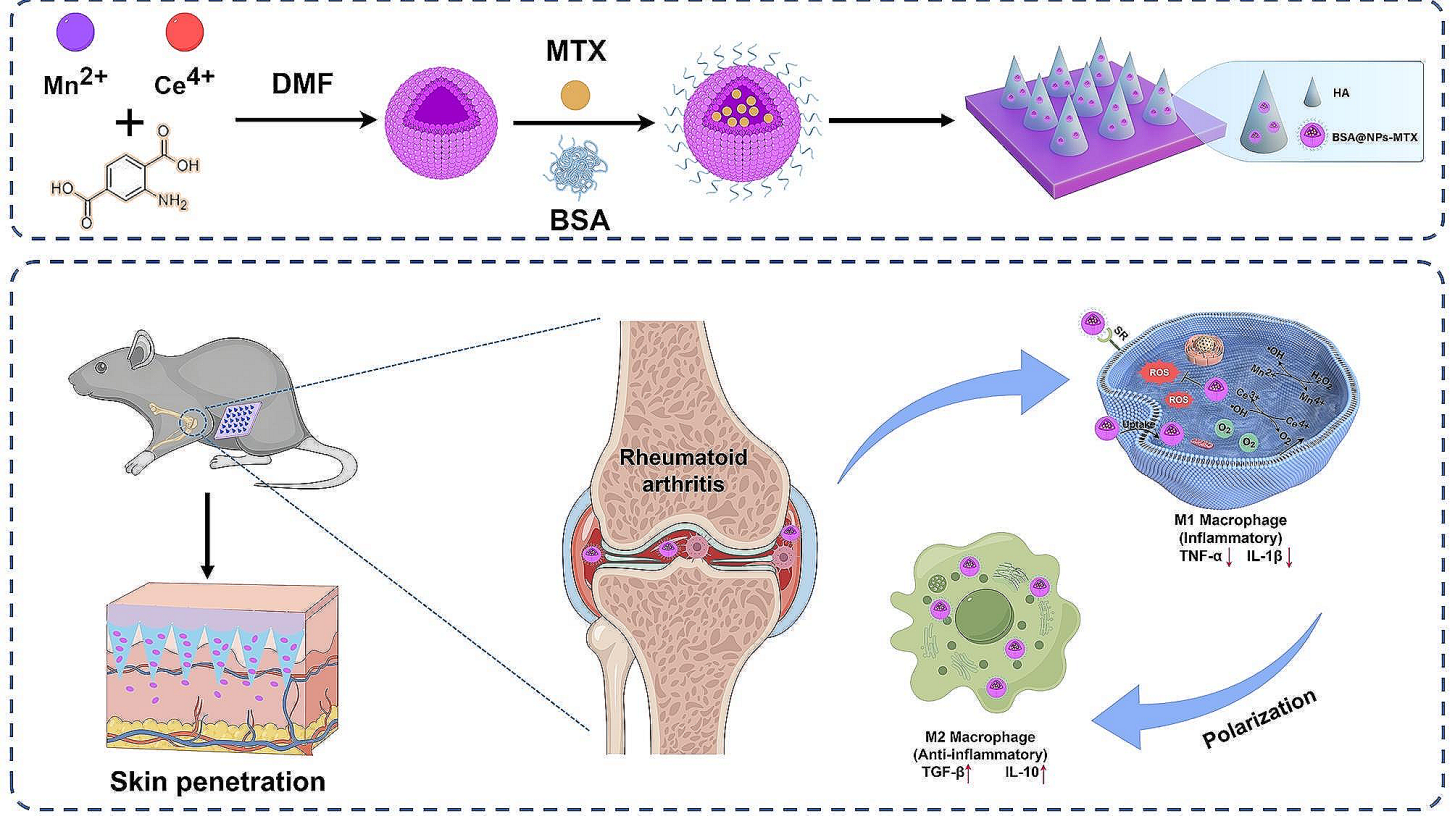
Schematic illustration of BSA@NPs-MTX preparation and corresponding therapeutic mechanism in RA treatment. Figure was created by Figdraw

In this study, we constructed a minimally invasive programmable MN platform [32] by mixing hyaluronic acid (HA) with BSA@NPs-MTX. With the degradation of MNs, the released BSA@NPs-MTX actively targeted M1 macrophages and reduced their production by clearing ROS and limiting the secretion of inflammatory factors. This simultaneously improved the hypoxic conditions of the knee joint, promoted the polarization of M2 macrophages, regulated the M1/M2 macrophage balance, and effectively delivered MTX for RA treatment (Scheme 1). This innovative approach not only provides an advanced drug delivery system but also highlights the potential of MN-assisted transdermal delivery combined with nanotechnology to address the complexity of RA. The BSA@NPs-MTX delivery system demonstrates regulation of macrophage polarization and clearance of ROS, showing great potential for the treatment of immune-related diseases.

## Materials and methods

### Materials

Manganese chloride tetrahydrate and ammonium ceric nitrate were obtained from Aladdin (Shanghai, China). Polyvinylpyrrolidone (PVP) and N, N-dimethylformamide (DMF, ≥ 99.5%) were obtained from Macklin (Shanghai, China). 2′,7′-Dichlorofluorescin diacetate (DCFH-DA) was provided by Beyotime (Shanghai, China). Dulbecco’s modified eagle medium (DMEM), penicillin, streptomycin, trypsin and cell counting kit-8 (CCK-8) were purchased from Meilunbio (Dalian, China). Foetal bovine serum (FBS) was obtained from Gibco (Guangzhou, China). Anti-CD86, anti-CD206, anti-CD3, anti-CD4, F4/80, and Foxp3 were obtained from BioLegend (San Diego, CA, USA). Lipopolysaccharide (LPS) and Interferon-γ (IFN-γ) were purchased from Sigma-Aldrich (Santa Barbara, CA, USA). The hematoxylin and eosin (HE) staining kit, catalase (CAT) activity assay kit, and superoxide dismutase (SOD) activity assay kit were purchased from Solarbio (Beijing, China). All chemicals and reagents were used as received without additional purification. All solutions were prepared with deionised (DI) water, which was purified through an 18-MΩ system (Millipore, USA).

### Preparation and characterization of BSA@NPs-MTX

According to a previously reported method [33], DMF (9 mL) and methanol (1.2 mL) were firstly added to a three-neck flask. Secondly, 0.08 g of 2-aminoterephthalic acid, 0.12 g of cerium ammonium nitrate, 0.22 g of manganese chloride tetrahydrate, and 0.25 g of PVP were added in sequence. The mixed solution was then transferred to a 50 mL stainless-steel autoclave lined with polytetrafluoroethylene and heated in a vacuum drying oven at 150 °C for 4 h. After the reactor cooled to room temperature, the solution was centrifuged at 11,000 × rpm for 5 min, and the resulting precipitate was washed once with DI water and once with anhydrous ethanol. And then the supernatant was collected after centrifuging at 2000 × rpm for 3 min. Finally, the obtained sample was suspended in anhydrous ethanol.

1 mL of MTX solution (0.2 mg/mL in DI water) was combined with 1 mL of NPs and stirred overnight under ambient conditions. The unloaded MTX was removed by multiple washes with DI water. Subsequently, MTX-loaded NPs (NPs-MTX) were introduced into 1 mL of BSA solution (60 mg/mL) and allowed to react for 24 h under ambient conditions. Finally, the BSA@NPs-MTX complex was isolated by centrifugation and purified by three successive washes with DI water.

The morphology of NPs was observed using transmission electron microscopy (TEM; FEI Talos F200S G2, USA). The hydrodynamic size and zeta potential of the NPs were determined by dynamic light scattering (DLS; Zetasizer Nano ZS ZEN3600, Malvern, UK) at room temperature. Fourier transform infrared (FTIR) spectra were acquired using an FTIR spectrometer (Tensor II, Bruker, Germany), and the absorption spectrum of the NPs was documented using ultraviolet-visible-near-infrared (UV-vis-NIR) spectrophotometry (CARY 5000, USA). To evaluate the loading capacity of MTX, BSA@NPs-MTX was dissolved in 2 mL of 1 × PBS solution. Following agitation and centrifugation, the resultant supernatant was isolated to quantify the residual MTX. The loading capacity and efficiency of MTX incorporation were ascertained using UV-vis-NIR spectrophotometry.

To measure the in vitro release profile of MTX in BSA@NPs-MTX, 5 mL of BSA@NPs-MTX was dialysed against 30 mL of PBS (pH 7.4) and incubated at 37 °C with constant stirring at 120 × rpm. Periodically, 5 mL of the surrounding buffer was extracted and promptly replenished with fresh PBS. The MTX concentrations in these aliquots were quantified using UV-vis-NIR spectrophotometry.

### Antioxidant properties of the NPs

Briefly, different concentrations of NPs were incubated with 2,2′-amino-di (2-ethylbenzothiazoline sulphonic acid-6) ammonium salt (ABTS) and 2,2-diphenyl-1-picrylhydrazyl (DPPH) for 30 min in darkness [34]. Subsequently, the absorbances at the characteristic wavelengths of ABTS (734 nm) and DPPH (517 nm) were quantified using a UV-vis-NIR spectrophotometer. The ABTS and DPPH scavenging rates were calculated.

A hydroxyl free radical-scavenging capacity assay kit was used to assess the hydroxyl radical-scavenging capabilities of the NPs. The Fenton reaction, initiated by H_2_O_2_/Fe^2+^, produces hydroxyl radicals that subsequently oxidise Fe^2+^ to Fe^3+^ within the orthophenanthroline-Fe^2+^ aqueous solution, decreasing the absorbance at 536 nm. The ability to scavenge hydroxyl radicals is related to the absorbance at 536 nm [35].

To evaluate the H_2_O_2_ neutralised by the NPs, various concentrations of NPs were incubated in 2 mL of PBS solution fortified with 400 µM H_2_O_2_ at 37 °C for 24 h. The residual H_2_O_2_ concentration was determined using the H_2_O_2_ detection kit by measuring the absorbance at 405 nm, from which the H_2_O_2_ scavenging proficiency was derived [36].

In addition, the ability of NPs to neutralise superoxide anions was evaluated. In the designated assays, different NPs concentrations were exposed to superoxide anions. The residual superoxide anion concentration was determined using a superoxide anion-scavenging capacity assay kit. Superoxide anions oxidised hydroxylamine hydrochloride to produce nitrite. Upon interaction with aminobenzenesulfonic acid and α-naphthylamine, the nitrite formed a red azo compound, exhibiting a characteristic absorption peak at 530 nm. The absorbance at 530 nm was used for quantitative detection of superoxide anions.

The SOD-mimetic potential of the NPs was assessed using an SOD activity assay kit according to the manufacturer’s protocol. In essence, the superoxide radical anion (O_2_•^−^) was produced through the xanthine and xanthine oxidase reaction system. O_2_•^−^ reduced nitro blue tetrazolium to form blue formazan, which was absorbed at 560 nm. SOD catalysed the dismutation of O_2_•^−^ to produce H_2_O_2_ and O_2_, thereby inhibiting formazan formation. The deeper the blue colour of the reaction solution, the lower the activity of SOD. And the CAT mimetic potential of the NPs was assessed using a CAT activity assay kit according to the manufacturer’s protocol. The decomposition of H_2_O_2_ by the NPs was rapidly arrested using ammonium molybdate. The remaining H_2_O_2_ interacted with ammonium molybdate to yield a yellow compound. The absorbance of this compound was measured at 405 nm using spectrophotometry and served as an indicator of CAT activity.

### Cell viability and cellular uptake

RAW264.7 cells were cultured on circular microscope slides situated in the 24-well plates at a density of 2.0 × 10^5^ cells per well in DMEM supplemented with LPS (100 ng/mL) and IFN-γ (20 ng/mL). After incubation for 24 h, these cells were exposed to rhodamine B, NPs loading rhodamine B, and BSA@NPs loading rhodamine B for 4 h. After three washes with PBS, cells were fixed with 4% paraformaldehyde for 10 min and blocked with 1% BSA for an additional 30 min. Nuclear staining was accomplished with 4′,6-diamidino-2-phenylindole (DAPI) for 10 min. Fluorescence microscopy (Axio Vert.A1; Carl Zeiss, Oberkochen, Germany) was used to visualise the cells. Cells after the same treatment were isolated, rinsed three times with 1 × PBS, and prepared for flow cytometry.

### In vitro ROS scavenging

RAW264.7 cells were cultured on circular microscope slides within a 24-well plate at a density of 2.0 × 10^5^ cells per well in DMEM supplemented with LPS (100 ng/mL) and IFN-γ (20 ng/mL). After 24 h, the cells were exposed to various concentrations of NPs for another 24 h. Subsequently, the cells were stained with DCFH-DA (Beyotime, Beijing, China) at 37 °C for 30 min and with DAPI for 10 min. Cellular imaging was performed using an inverted fluorescence microscope. Concurrently, for flow cytometric assessments, RAW264.7 cells were placed in a 24-well plate at a density of 2.0 × 10^5^ cells per well. After 24 h exposure to various concentrations of NPs, the cells were stained with DCFH-DA, and ROS generation was quantified by flow cytometry.

### The effects of NPs on macrophages

RAW264.7 cells were cultured in a 24-well plate at a density of 2.0 × 10^5^ cells per well using a medium supplemented with LPS (100 ng/mL) and IFN-γ (20 ng/mL). The cells were incubated at 37 °C for 24 h before exposure to various formulations for another 24 h.

The phenotypic switching of macrophages was examined using flow cytometry. Following the treatment, the cells were harvested by trypsinisation and blocked with 1% BSA for 30 min. The cells were washed with PBS wash once and labelled with PE-conjugated F4/80 (BD pharmingen, USA), PB450-conjugated CD86 (Thermo Fisher Scientific, USA), and APC-conjugated CD206 (Thermo Fisher Scientific, USA) for 40 min at 4 °C. For APC-conjugated CD206 staining, the cells were fixed and permeabilised using the cytofix/cytoperm fixation/permeabilisation kit (BD Pharmingen, USA) prior to staining. Stained cells were washed with PBS supplemented with 1% FBS and analysed using a flow cytometer (CytoFLEX, USA). Immunofluorescence analysis was performed on the cells subjected to the same treatment regimen.

To further investigate the mRNA expression of M1 and M2 macrophage markers, the cells were subjected to various treatments for an additional 24 h. Total RNA was extracted by the RNA-Quick Purification Kit (Yishan Bio, China). The extracted mRNA was reverse-transcribed using a PrimeScript RT reagent kit (TaKaRa Bio, Shiga, Japan). Quantitative real-time polymerase chain reaction (qRT-PCR) was conducted to assess the mRNA levels using SYBR Premix Ex Taq (TaKaRa Bio) with specific primers. The expression levels of the target genes were standardised against that of the housekeeping gene GAPDH. The primer sequences for the genes of interest were listed in Table S1.

To evaluate anti-inflammation effects of NPs in vitro, the cells were subjected to various treatments for an additional 24 h. Thereafter, the concentrations of specific inflammatory cytokines, including TNF-α and IL-1β, in the culture supernatant were quantified using ELISA kits (Peprotech, Thermo Fisher Scientific, USA).

To assess the expression of HIF-1α, RAW264.7 cells were cultured in DMEM supplemented with LPS and subjected to hypoxic conditions for 4 h. Then, cells were treated with a fresh medium containing 100 µg/mL of NPs for an additional 4 h. Following the treatment, cells were fixed with paraformaldehyde and permeabilised with 0.1% Triton X-100. Blocking was achieved by incubating the cells in 10% serum for 45 min at room temperature. The cells were then probed with primary antibodies, namely anti-HIF-1α (BF8002) and anti-beta tubulin (AF7011), for 1 h at 37 °C. An AlexaFluor594-conjugated goat anti-mouse IgG (red) was used as a secondary antibody.

### Preparation and characterization of MNs

A polydimethylsiloxane (PDMS) mould procured from Taizhou Microchip Pharmaceutical Technology Co., Ltd. (Taizhou, China) with a length of 1 mm (array size: 15 × 15; bottom dimensions: 0.45 mm × 0.45 mm) was employed for MN fabrication. A 10% (w/v) HA solution was prepared by dissolving HA powder in DI water. Then, MTX and BSA@NPs were added to the HA solution. The mixture was introduced into a PDMS mould, followed by centrifugation at 5000 × rpm for 5 min. The mould was then desiccated at an ambient temperature for 24 h. A 25 wt% PVP aqueous solution was subsequently added to the mould and centrifuged at 5000 × rpm for 5 min to construct the base layer. The PDMS mould was carefully removed to retrieve MNs, and the MNs were stored at ambient temperature for further analysis. MNs loaded with rhodamine B were synthesised using a similar method. The architecture and morphology of the MNs were examined by field-emission scanning electron microscopy (FE-SEM; SU8010, HITACHI, Japan), optical microscopy, and confocal laser scanning microscopy (CLSM; Nikon A1, Japan).

To determine the drug content within the MNs, the MNs were separated and dissolved in PBS. The absorbance of the resulting solution was quantified using a UV-vis-NIR spectrophotometer at 302 nm, and the MTX concentration was deduced from a reference standard curve.

The mechanical properties of the MNs were assessed using an electronic universal material-testing machine (Instron 5944). The preliminary gap between the MN tips and the upper plate was 2 mm, and the sensor motion speed for the top plate was 0.01 mm/s. The acquired force and displacement parameters were subsequently plotted to generate a force-displacement graph.

### Skin penetration test in vivo

The skin penetration efficacy of the MNs was evaluated in murine skin. Before applying MNs, the dorsal region of the mouse was depilated under anaesthesia. After 5 min, the MN array was carefully removed, and the treated skin area was visualised using an optical microscope. The skin sample was excised, preserved in 4% paraformaldehyde for 24 h, processed for embedding, sectioned, and subsequently stained with hematoxylin and eosin (H&E). The histological examination of skin penetration was performed using an Olympus CKX53 microscope.

### In vivo dissolution of MNs

The HA MNs were applied to the hairless dorsal skin and subsequently removed at specific intervals (0, 3, 5, 10, 15, and 20 min). After removal, the MNs were sectioned into strips. The dissolution of the MNs were imaged under an Olympus SZ61 microscope. The dissolution kinetics was represented by plotting the residual height percentages of the HA MNs at specified time points.

### In vitro drug release

MNs were submerged in PBS in centrifuge tubes, and the tubes were subsequently incubated at 37 °C with agitation. At specific time points, 1 mL of the suspension was replaced with an equivalent volume of fresh PBS. The drug release profiles from the MNs were quantified using a UV-vis-NIR spectrophotometer.

For in vitro transdermal absorption studies, a Franz diffusion cell apparatus with a 10 mL receptor chamber was employed. The MNs were embedded in Parafilm M, and the receptor chamber was filled with 10 mL of PBS (pH 7.4) as the receiving medium. At the predetermined time points, 1 mL of the solution was extracted and replaced with a fresh aliquot of PBS. The drug concentrations in the solutions were assessed using a UV-vis-NIR spectrophotometer at a wavelength of 302 nm.

### Therapeutic efficacy evaluations in vivo

All experimental procedures involving animals followed the guidelines stipulated in the Guidance Suggestions for the Care and Use of Laboratory Animals. The study protocol was approved by the Animal Research and Ethics Committee of the Wenzhou Institute of the University of the Chinese Academy of Sciences (Approval Reference: WIUCAS23020207). Mice were maintained in a specific pathogen-free facility at 25 °C, following a 12-h light/dark regimen.

Male DBA/1 mice aged 6–8 weeks were purchased from Beijing Vital River Laboratory Animal Technology Co. Ltd. (Beijing, China). The initial immunisation involved intradermal injection of an equal-volume mixture of bovine type-II collagen solution (2 mg/mL) and complete Freund’s adjuvant (4 mg/mL) into the tail base. Booster immunisation was administered after 21 d using bovine type-II collagen solution emulsified in incomplete Freund’s adjuvant to intensify disease manifestation.

After the second immunisation, the collagen-induced arthritis (CIA) model mice were randomly divided into five groups (*n* = 6): normal group, model group, MTX MNs group, BSA@NPs MNs group, and BSA@NPs-MTX MNs group. Mice were treated every two days, and the clinical scores and paw thickness were observed and recorded. After 20 d of treatment, the mice were sacrificed, and their hind limbs were collected. After fixation in 4% paraformaldehyde, the knee joints and paws were imaged with Micro‑computed tomography (micro-CT; Bruker, Germany). Bone parameters, including bone surface area to bone volume ratio (BS/BV), bone volume to tissue volume ratio (BV/TV), and trabecular number (Tb.N), were quantitatively assessed using proprietary analysis software (CTAn, Version 1.15.4.0, Germany). Serum concentrations of TNF-α, IL-1β, interleukin-6 (IL-6), interleukin-10 (IL-10), and transforming growth factor-β (TGF-β) were quantified utilising ELISA assays. Histopathological analyses of knee specimens included H&E staining, safranin-O staining, immunohistochemical staining for TNF-α and IL-1β, and immunofluorescent staining targeting CD86, CD206, and HIF-1α. Concurrently, key mouse organs were subjected to histological examination using H&E staining.

Histopathological evaluations were conducted to delineate the characteristic RA markers, including hypoxia, M1/M2 macrophage polarization, synovial inflammation, and cartilage degradation. Knee joint specimens were preserved in 4% paraformaldehyde and decalcified in a solution from Elabscience (Wuhan, China) with agitation for 28 d at ambient temperature. The decalcified tissues were then embedded in paraffin and sectioned into 10 μm slices using a microtome.

For immunohistochemical analysis, sections were dewaxed and incubated with primary antibodies against HIF-1α (Abcam), CD86 (ABclonal), and CD206 (Abcam). Subsequently, secondary antibodies were applied, including goat anti-rabbit Alexa Fluor 568 (Abcam) for HIF-1α and CD86 and goat anti-rabbit Alexa Fluor 488 (Abcam) for CD206. After antibody incubation, sections were counterstained with DAPI mounting medium and visualised by CLSM.

### Statistical analysis

Data analysis was performed using GraphPad Prism 8 software (GraphPad Software, USA). The results were presented as mean ± standard error of the mean. Differences among multiple groups were determined using one-way analysis of variance (ANOVA). Comparisons between two distinct groups were performed using unpaired two-tailed t-tests. The statistical significance thresholds were set at *p* < 0.05 (*), 0.01 (**) and 0.001 (***).

## Results and discussion

### Preparation and characterisation of NPs

**Fig. 1:**
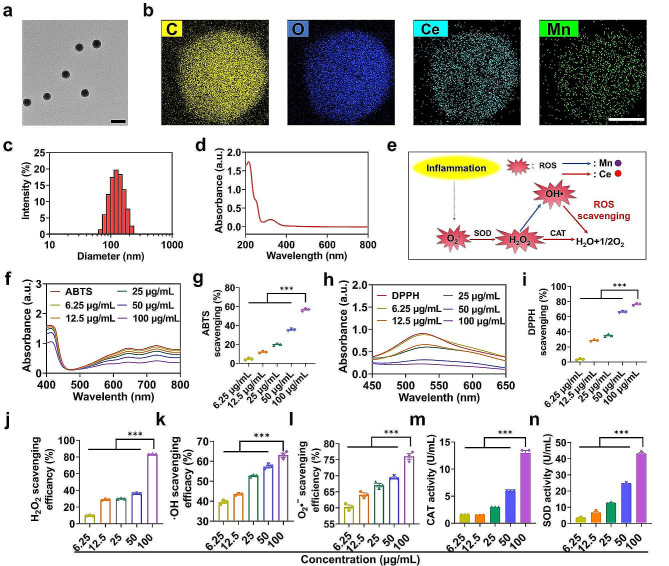
Characterization of NPs. (**a**) Representative TEM image of NPs. Scale bar:200 nm. (**b**) STEM image of NPs and corresponding elemental mapping images of C, O, Ce and Mn of NPs. Scale bar: 50 nm. (**c**) Size distribution of the NPs. (**d**) UV–vis absorbance spectrum of the NPs. (**e**) Schematic illustration of the mechanism of ROS scavenging by NPs. (**f**) The concentration-dependent research of ABTS in the presence of NPs. (**g**) Quantitative analysis of ABTS scavenging capabilities after incubation with different concentrations of NPs. (**h**) UV-vis spectra of DPPH after incubation with various concentrations of NPs as mentioned. (**i**) Quantitative analysis of DPPH scavenging capabilities. (**j**) The H_2_O_2_ scavenging efficiency of NPs. (**k**) The hydroxyl radical (·OH) scavenging efficiency of NPs. (**l**) Superoxide anion (O_2_•^−^) scavenging activity of the NPs at different concentrations. (**m-n**) Evaluation of (m) CAT and (n) POD-like activities of NPs at different concentrations. Data were expressed as the mean ± SEM (*n* = 3, *p < 0.05, **p < 0.01, ***p < 0.001)

In this study, NPs were synthesized using a simple one-pot biomimetic mineralisation method. The TEM image in Fig. 1a elucidated that the NPs were monodisperse spheres with a mean diameter of approximately 100 nm. Elemental mapping via energy-dispersive X-ray spectroscopy (EDS) detected C, O, Ce, and Mn, confirming the successful synthesis of the NPs (Fig. 1b). DLS data showed that the hydrodynamic diameter of the NPs was approximately 117.4 nm, and the polydispersity index (PDI) was 0.088 (Fig. 1c). The UV-vis-NIR spectrum of the NPs showed a salient absorption peak at 320 nm (Fig. 1d).

Recent reports have shown that silver nanoparticles and albumin-cerium oxide nanoparticles are used for the treatment of RA. However, the catalytic activity of single metal was insufficient, while the synergistic effect of mixed metal ions demonstrates excellent capabilities in catalyzing H_2_O_2_ and eliminating ROS [37, 38]. Therefore, we designed cerium/manganese oxide nanoparticles to treat RA in this study. Mn^2+^ catalysed the conversion from H_2_O_2_ to •OH, whereas Ce^4+^ transformed hydroxyl free radicals, which were intermediates of the Fenton reaction, into O_2_ molecules. Such Mn/Ce-mediated synergistic effect could amplify Fenton chemistry to facilitate ROS eradication in RA knee joints and generate O_2_ to promote the polarization of macrophages from M1 to M2, thus alleviating inflammation (Fig. 1e). Initially, the antioxidant potential was ascertained using the ABTS free radical-scavenging assay. As depicted in Fig. 1f, a notable reduction was observed in the ABTS absorbance at 734 nm after co-incubation with NPs. A quantitative analysis revealed that the free radical-scavenging ability was concentration-dependent, with the maximum scavenging ability at 100 µg/mL NPs (56.68 ± 1.53%), as shown in Fig. 1g. Figure 1h showed a significant drop in DPPH absorption at 517 nm post-NPs administration. The 100 µg/mL NPs exhibited a DPPH radical-scavenging capacity of 76.44 ± 1.53%, twenty times that of 6.25 µg/mL and twice that of 25 µg/mL, as depicted in Fig. 1i. Subsequently, typical bio-relevant ROS species, i.e., H_2_O_2_, •OH, and O_2_•^−^, were used to investigate the ROS-scavenging efficacy of NPs at various concentrations [39]. As expected, the NPs effectively eliminated H_2_O_2_, •OH, and O_2_•^−^ species, as evidenced by the dose-dependent trends observed post-incubation (Fig. 1j-l). At 100 µg/mL of NPs, the clearance rates for H_2_O_2_, •OH, and O_2_•^−^were 83.16 ± 0.30%, 63.10 ± 1.85%, and 76.07 ± 1.53%, respectively. Ceria nanoparticles have been reported as effective antioxidants with robust multiple ROS-scavenging capabilities. Their catalase mimetics and superoxide dismutase mimetics are attributed to Ce (IV) and Ce (III) sites, respectively [37, 38]. In this study, the enzymatic mimetic activity of NPs was investigated. CAT catalysed the decomposition of H_2_O_2_ into H_2_O and O_2_, thereby preventing the accumulation of H_2_O_2_ and protecting organisms from oxidative damage induced by H_2_O_2_. The decomposition rate of H_2_O_2_ depended on the increased concentrations of NPs, reaching 12.96 U/mL at a concentration of 100 µg/mL NPs, as illustrated in Fig. 1m, thus confirming that NPs could serve as mimetic catalases to clear H_2_O_2_. SOD catalysed the conversion of superoxide anion radicals into H_2_O_2_ and O_2_ and played a crucial role in defending against cellular damage caused by oxygen-free radicals. As depicted in Fig. 1n, as the concentration of NPs increased, SOD activity gradually increased. Collectively, these results indicated that the constructed NPs exhibited multi-enzymatic mimetic activity and could effectively clear various types of ROS.

### Effect of NPs on ROS scavenging and targeting M1-type macrophages in vitro

**Fig. 2:**
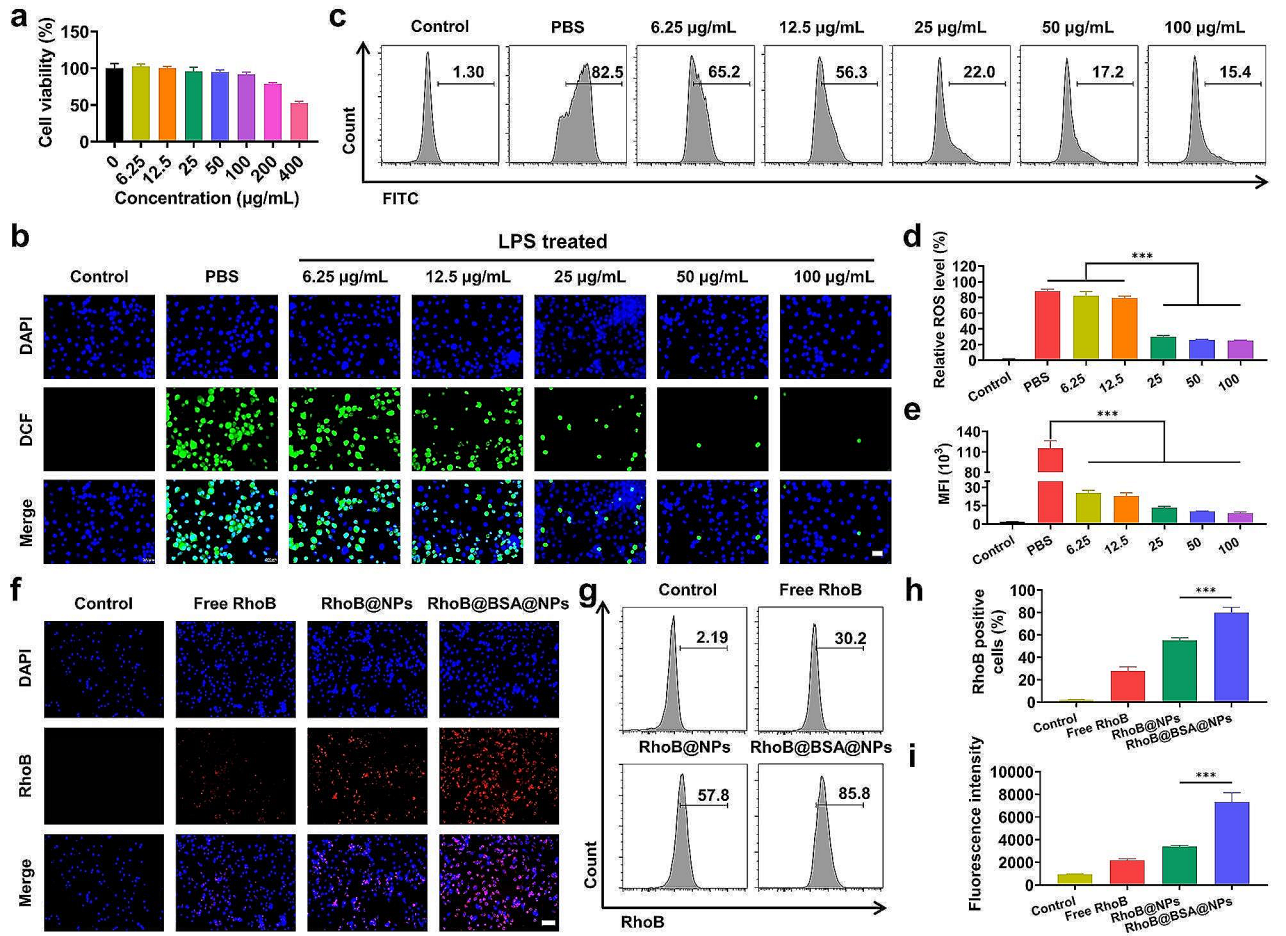
In vitro ROS scavenging and capability to target M1 macrophages by NPs. (**a**) Cell viability of RAW264.7 cells after incubation with NPs at various concentrations for 24 h as indicated. (**b**) Representive fluorescence microscopy images of intracellular DCFH-DA (green) in LPS-activated RAW264.7 cells treated with different concentrations of NPs for 24 h. Scale bars: 50 μm. (**c-e**) DCF fluorescence signals and mean fluorescence intensity determined by flow cytometry analysis after 24 h incubation. (**f**) Fluorescence microscopy observed of the Intracellular uptake of NPs in RAW264.7 cells. Nuclei were stained with DAPI (blue) and NPs were labeled using rhodamine B (red). Scale bars: 50 μm. (**g-i**) Intracellular fluorescence signals were determined by flow cytometry analysis after 4 h incubations with rhodamine B-labeled NPs and BSA@NPs. Data were expressed as the mean ± SEM (*n* = 3, *p < 0.05, **p < 0.01, ***p < 0.001)

The cytotoxicity of NPs to RAW264.7 was first assessed to ensure their biosafety. As shown in Fig. 2a, cell viability consistently exceeded 90% after exposure to NPs for 24 h, even for the highest tested concentration (100 µg/mL). These findings indicated that NPs had low cytotoxicity to RAW264.7 cells.

To determine the ability of NPs to scavenge intracellular ROS, a DCFH-DA probe was utilized to label ROS in RAW264.7 cells cultured with various concentrations of NPs in the presence of LPS (100 ng/mL). Immunofluorescence staining showed that cells in the PBS group exhibited a strong fluorescence, suggesting that inflammatory mediators induced ROS production in macrophages and triggered a strong inflammatory response. In contrast, a weak fluorescent signal was observed in the control and NPs-treated groups, and the fluorescence decreased with the increasing NPs concentration (Fig. 2b). As shown in Fig. 2c-e, flow cytometry quantification suggested that 25 µg/mL of NPs had good scavenging ability. As the concentration of NPs increased, the level of ROS in macrophages decreased, confirming that the NPs effectively inhibited ROS production in macrophages. Furthermore, quantification of the immunofluorescence intensity indicated that the NPs could effectively clear ROS (Figure S1).

The HIF-1α expression levels were measured to verify the hypoxia-alleviating capability of NPs. RAW264.7 cells were incubated under hypoxic and inflammatory conditions for 4 h, followed by co-incubation with 100 µg/mL of NPs for 24 h. After the incubation, the intracellular HIF-1α levels were observed using CLSM. Hypoxia and LPS upregulated the expression of HIF-1α, but the fluorescence was weakened, suggesting that NPs could produce oxygen to alleviate cellular hypoxia while they scavenged hydroxyl radicals (Figure S2).

The targeting of BSA to M1-type macrophages was studied using flow cytometry and inverted fluorescence microscopy [25, 40]. RAW264.7 cells were polarised into M1-type macrophages (M1-Mø) in the presence of inflammatory factors LPS and IFN-γ. To visualise the cellular uptake levels, fluorescence images were utilized to localise the fluorescent NPs within RAW264.7 cells. Cell nuclei were stained with DAPI (blue), and rhodamine B (red) fluorescent dye was utilized to label NPs and BSA@NPs, which were subsequently co-cultured with M1-Mø for 4 h. After incubation, cells were rinsed and examined under an inverted fluorescence microscope. A conspicuous red fluorescence was discerned in RAW264.7, signalling the internalisation of rhodamine B-labelled NPs into the cellular matrix. The M1-Mø cells treated with BSA@NPs had significantly higher fluorescence intensity than those treated with NPs (Fig. 2f), indicating that BSA promoted the uptake of nanoparticles by macrophages. The uptake levels and average fluorescence intensities of RAW264.7 cells for different NPs were quantified using flow cytometry. As depicted in Fig. 2g-i, the uptake of BSA@NPs was 1.45 times that of NPs, suggesting that BSA could specifically target scavenger receptors on the polarised RAW264.7 cells and promote cellular uptake.

### NPs regulated M1/M2 macrophage polarization

MTX has been widely used as a small-molecule DMARD for RA treatment [12]. In this study, MTX was encapsulated by NPs for extended release, aiming for enhanced treatment efficacy in an in vivo RA mouse model. The size of NPs-MTX was determined as 123.6 nm through DLS measurements, and the PDI of NPs-MTX was 0.281. The size of BSA@NPs-MTX was 144.8 nm. The zeta potentials of NPs, NPs-MTX, and BSA@NPs-MTX in H_2_O were approximately − 3.13 mV, -4.18 mV, and − 12.7 mV, respectively (Figure S3). To emulate the stability of NPs in physiological environments, the particle sizes of BSA@NPs-MTX in H_2_O, normal saline, and PBS buffer containing 10% FBS were examined. DLS analysis showed that the BSA@NPs-MTX particles remained 120–150 nm in these media with no significant change over seven days (Figure S4). As shown in Figure S5, the absorption spectrum of BSA@NPs-MTX encompassed the peaks of BSA (278 nm) and MTX (317 nm) with a shift caused by the NPs. Furthermore, the FTIR spectrum of MTX [32], as illustrated in Figure S6a, showed a band between 3300 and 3400 cm^− 1^ corresponding to the stretching vibration of N-H. The band at 1641 cm^− 1^ represented the -CONH group. Additionally, the band at 1601 cm^− 1^ was linked to the C = C stretching vibration inherent in the benzene ring. The 1496 cm^− 1^ band correlated with the symmetric stretching mode of COO-. Finally, the peaks around 1208 cm^− 1^ signified C-N stretching vibrations. Notably, distinctive MTX peaks were observed in BSA@NPs-MTX spectrum (Figure S6b). As a rapid release of MTX could cause first-pass effect and cytotoxicity, a sustained release at a reduced rate was advantageous for RA treatment. The recorded MTX loading amount and efficiency were maintained at 41.2 µg/mg and 27.2%, respectively. The MTX release profile revealed a gradual release of approximately 60% within six days (Figure S7). As shown in Figure S8, BSA@NPs-MTX exhibited low toxicity to cells.

**Fig. 3:**
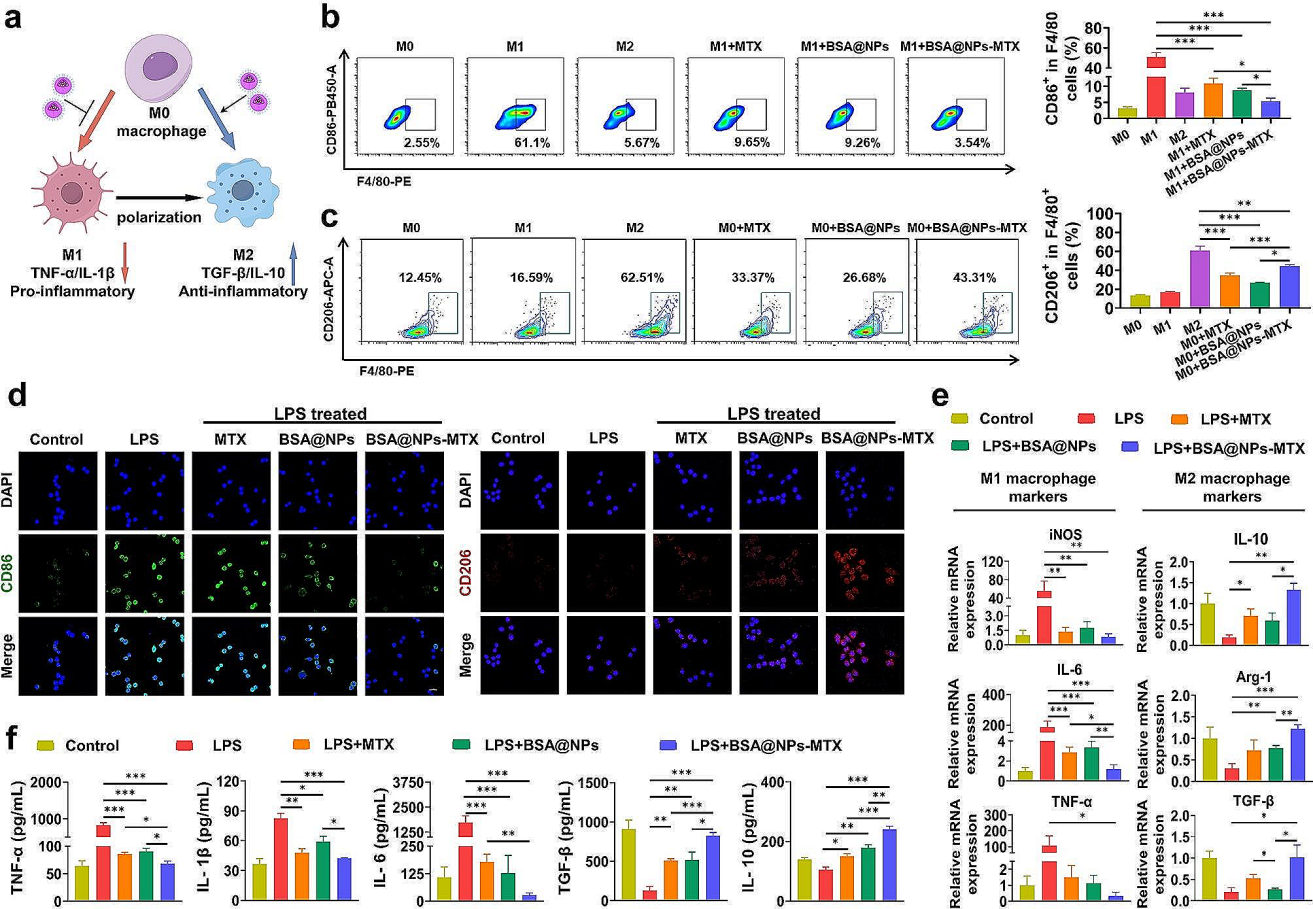
Macrophage phenotype regulation based on BSA@NPs-MTX in vitro. (**a**) The schematic illustration of BSA@NPs-MTX regulated macrophage polarization under LPS. (**b-c**) The flow cytometric analysis and quantification results of RAW264.7 (gated on F4/80^+^) incubated with different formulations in LPS conditions. (**d**) The immunofluorescence images of RAW264.7 cultured with different formulations under LPS conditions. CD86^+^ (green) M1 macrophage and CD206^+^ (red) M2 macrophage, nuclei (DAPI: blue). Scale bar: 50 μm. (**e**) The relative mRNA expression of M1 and M2 macrophage markers in RAW264.7 cells after incubation with different conditions, as assessed by qRT-PCR analysis. (**f**) Expression factors of iNOS, IL-6, TNF-α, IL-10 and Arg-1 as well as TGF-β in LPS-activated RAW264.7 cells after treating with various formulations, as measured by Elisa. Data were expressed as the mean ± SEM (*n* = 3, *p < 0.05, **p < 0.01, ***p < 0.001)

The polarization of macrophages treated with BSA@NPs-MTX was also examined (Fig. 3a). Based on the literature, M1-type and M2-type macrophages had a higher expression of F4/80^+^CD86^+^ and F4/80^+^CD206^+^, respectively [34]. Flow cytometry analysis showed that the average level of F4/80^+^CD86^+^ M1 macrophage was decreased from 51.05 ± 8.60% to 5.41 ± 1.87% after co-incubation with BSA@NPs-MTX treatment for 24 h (Fig. 3b). Moreover, BSA@NPs-MTX treatment increased the percentage of F4/80^+^CD206^+^ M2 macrophage from 16.77 ± 1.44% to 44.44 ± 2.55% compared with PBS treatment (Fig. 3c). Similar findings were observed in the MTX and BSA@NPs groups. These results further indicated that BSA@NPs-MTX could inhibit the inflammatory response and enhance M2 polarization. Immunofluorescence images and quantification showed the same trend (Fig. 3d, S9).

qRT-PCR and ELISA were utilized to explore the effects of BSA@NPs-MTX on the secretion profile of macrophages. The qRT-PCR results indicated that LPS treatment increased the gene expression levels of M1 markers, including inducible nitric oxide synthase (iNOS), IL-6, and TNF-α. BSA@NPs-MTX treatment significantly reduced the mRNA expression levels of the M1 markers. However, after treatment with BSA@NPs-MTX, the anti-inflammatory M2 markers (arginase (Arg) -1, TGF-β, and IL-10) were significantly elevated (Fig. 3e). Macrophage polarization effects in MTX and BSA@NPs were less significant than that in BSA@NPs-MTX. Moreover, the levels of inflammatory cytokines in macrophages treated with BSA@NPs-MTX were examined. Comparing to the LPS group, the pro-inflammatory cytokine levels of IL-6, TNF-α, and IL-1β in the MTX, BSA@NPs, and BSA@NPs-MTX groups decreased, while the levels of anti-inflammatory cytokines TGF-β and IL-10 increased (Fig. 3f). To investigate the effect of BSA@NPs-MTX on chondrocytes, NPs were first co-cultured with macrophages. Subsequently, chondrocytes (pre-treated with H_2_O_2_) were added. After 24 h of co-cultivation, representative fluorescence images and quantitative analyses of cartilage formation were obtained. As shown in Figure S10, BSA@NPs-MTX increased the expression of type-II collagen in chondrocytes under oxidative stress. Collectively, these results revealed that BSA@NPs-MTX could modulate the articular inflammation and regulate the systemic immunity by shifting the balance of M1/M2 facilitating to the treatment of RA.

### Fabrication and characterization of MNs

**Fig. 4:**
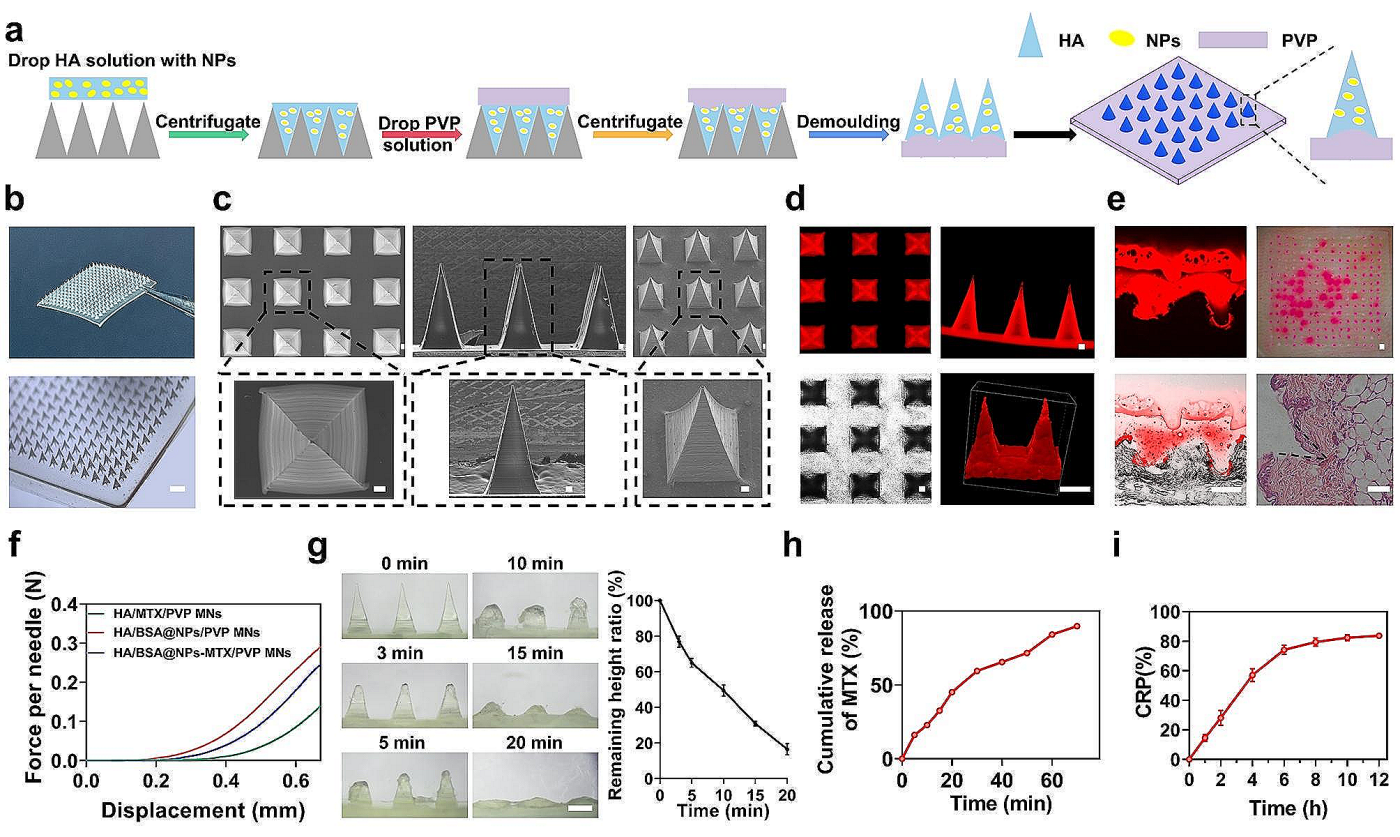
Fabrication and characterization of HA/PVP/MN. (**a**) Schematic of the fabrication process of MNs. (**b**) Digital images of HA/PVP MNs. Scale bar: 1 mm. (**c**) SEM images of side elevation at low and high magnification, vertical view of MNs at low and high magnification. Scale bar: 100 μm. (**d**) Fluorescence microscopy imaging of MNs with RhoB-labelled. Scale bar: 200 μm. (**e**) Fluorescent images of histological sections (Scale bar: 20 μm), photographs of mouse skin after the insertion of RhoB-loaded MNs (Scale bar: 1 mm), and H&E staining histological section images obtained by application of MNs (Scale bar: 500 μm). (**f**) The force–displacement curves of HA/MTX/PVP MNs, HA/BSA@NPs/PVP MNs and HA/BSA@NPs-MTX/PVP MNs. (**g**) Microscope images of MNs after insertion for different time and corresponding dissolution curves in vivo. Scale bar: 500 μm. (**h**) In vitro MTX release curves of MNs in PBS solution. (**i**) Skin drug release profile of MNs in vitro. Data were expressed as the mean ± SEM (*n* = 3, *p < 0.05, **p < 0.01, ***p < 0.001)

The cytotoxicity of MNs was measured (Figure S11). Initially, the HA solution was applied to the PDMS MN mould to form needle tips. Subsequently, the PVP solution was dispensed onto the mould, forming a soluble back patch (Fig. 4a). Figure 4b presented the digital microscopic images of the fabricated MN patches with a flexible back and a uniform MN array. The SEM image of the prepared MNs (Fig. 4c) revealed a pyramidal contour, which was further corroborated by their stereomicroscopic images. Each constructed MN patch comprised 225 (15 × 15) needles; each needle had an approximate width of ∼ 300 μm and a height of ∼ 760 μm, and the distance between adjacent needles was ∼ 400 μm. To observe the geometric structure of the MNs under CLSM, rhodamine B was used to stain the needle tip (Fig. 4d).

### Skin penetration and mechanical properties

Both insertion experiments were performed to confirm that the MNs successfully penetrated the skin in vitro and in vivo. Figure 4e presented a fluorescence micrograph of the tissue section after the administration of MNs infused with rhodamine B. The pronounced red fluorescence surrounding the penetration site validated the potential for drug dispersion into the dermal tissue. In the in vitro experiment, the MNs loaded with rhodamine B penetrated the separated dorsal skin of the mice for 5 min and were then removed. Red dots were observed on the isolated skin using a stereo microscope, demonstrating their puncturing capability. In the in vivo experiment, histological sections stained with H&E confirmed that the MNs penetrated the skin. Finally, these results indicated that MNs demonstrated superior penetration capabilities in the investigation and were essential for efficient transdermal drug delivery.

The mechanical properties of the prepared MNs were evaluated by compression tests. Figure 4f showed the force-displacement graph for the HA/MTX/PVP, HA/BSA@NPs/PVP, and HA/BSA@NPs-MTX/PVP MNs. The HA/MTX/PVP MNs were able to withstand compressive forces of 0.12 N per needle with a displacement of 600 μm. Notably, the HA/BSA@NPs/PVP MNs exhibited a high mechanical strength of 0.25 N per needle with a displacement of 600 μm. Previous research findings indicated that the minimal force necessary for skin penetration amounted to less than 0.1 N per needle [41]. UV-Vis spectroscopy was utilized to determine the MTX loaded in the MNs based on a standard curve (Fig. S12a). When the MTX concentration was 10 mg/mL, the MTX content in the MN tips was 91.5 µg (Figure S12b). As shown in Fig. 4g, morphological changes were observed in HA/PVP MNs at 0, 3, 5, 10, 15, and 20 min after skin insertion. The sharp edges before insertion were rounded within 10 min after insertion and disappeared after 20 min.

To assess the drug release profile, the MTX cumulative release percentage (CRP) in the MNs was measured in a PBS solution. As depicted in Fig. 4h, MTX rapidly released from the MNs within 20 min. For a more precise representation of the skin permeation and diffusion of the drug, an in vitro skin drug release assay was performed using a Franz diffusion cell. Figure 4i showed that approximately 75% of MTX was discharged from the MNs within 6 h post-application, delivering the drugs sustainably through the micropores generated by MNs penetration. As shown in Figure S13, after removing the MNs from the skin, the puncture marks progressively diminished. After 30 min, the skin returned to its normal state, and no obvious irritation was observed, indicating that MNs was a minimally invasive drug delivery platform.

### The therapeutic effects of MNs in CIA model

**Fig. 5:**
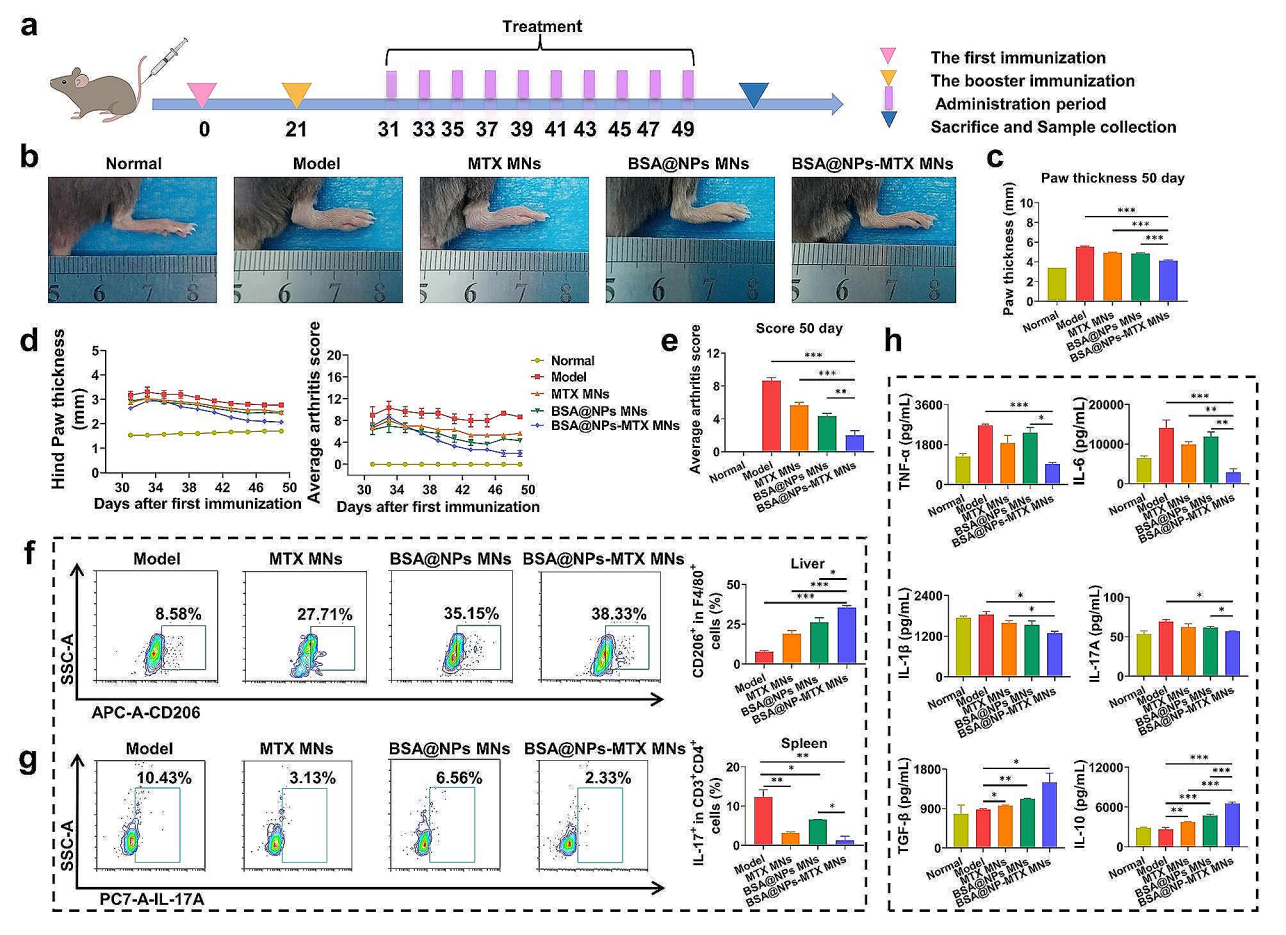
Therapeutic effect of the MNs in vivo. (**a**) Schematic illustration of the CIA mouse model establishment and treatment protocol. (**b**) Representative macroscopic hind paw images of each group after different formulations. (**c**) The average of hind paw thickness in mice on day 50. (**d**) Changes of hind paw thickness and arthritis scores in mice after different treatments. (**e**) The average of arthritis scores in mice on day 50. (**f**) Flow cytometry showed the percentage of F4/80^+^CD206^+^ M2 in F4/80^+^ macrophages in liver of CIA mice after different treatments. (**g**) Representative flow cytometric plots indicated the percentage of CD3^+^CD4^+^IL-17 A^+^ Th17 in spleen of CIA mice after different treatments. (**h**) ELISA measured cytokines levels in the serum of mice after different treatments. Data were expressed as the mean ± SEM (*n* = 6, *p < 0.05, **p < 0.01, ***p < 0.001)

The CIA model was utilized to evaluate the potential impact of NPs on the RA process. As shown in Fig. 5a, after the first immunisation on day 0 and the second immunisation on day 21, MNs treatments began on day 31 with administration every two days, and the mice were euthanised on day 50. As expected, mice in the model group treated with HA/PVP MNs showed significant redness and swelling around the paws (Fig. 5b). In contrast, the hind paw thickness of mice treated with MTX MNs and BSA@NPs MNs was significantly reduced, with the lowest thickness observed in the BSA@NPs-MTX MNs group (Fig. 5c). Consistent with the hind paw thickness, the arthritis score in the BSA@NPs-MTX MNs group also decreased significantly compared to those in the model group (Fig. 5d-e). These results indicated that BSA@NPs-MTX could effectively inhibit the development of arthritis.

To further explore the effects of NPs on the immunotherapy of RA mice, flow cytometry was utilized to detect the expressions of macrophages in the liver and Th17 cells in the spleen in each group. As shown in Fig. 5f, the level of anti-inflammatory F4/80^+^CD206^+^ M2 macrophage in liver was significantly upregulated in the BSA@NPs-MTX MNs group compared to that in the other groups, which was consistent with results of the hind paw thickness and arthritis score. After BSA@NPs-MTX MNs treatment, the percentage of Th17 cells was markedly reduced in spleen compared to that in model groups due to the inhibitory effect of MTX [42–45] on Th17 cell activity and proliferation (Fig. 5g, S14). Subsequent tests on mouse serum which was served as an indicator for assessing potential systemic immune responses, revealed that the treatment inhibited the expression of pro-inflammatory cytokines (TNF-α, IL-6, IL-1β, and IL-17) and promoted the secretion of anti-inflammatory cytokines (TGF-β and IL-10) (Fig. 5h). These results suggested that BSA@NPs-MTX MNs could alleviate RA inflammation and progression by regulating the immune environment in mice.

**Fig. 6:**
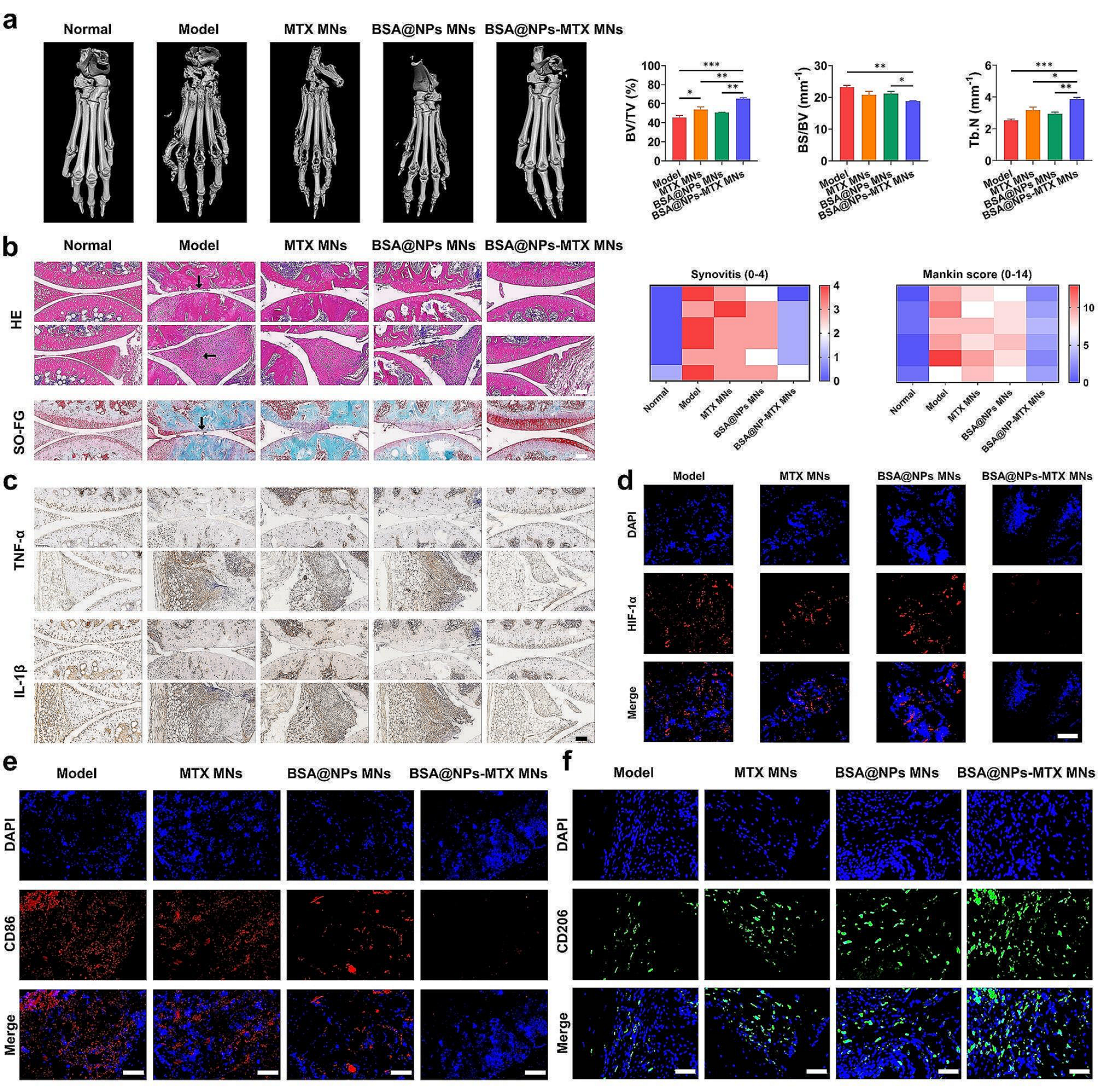
The effect of MNs on joint injure and inflammatory response in RA model. (**a**) Micro-CT images of the hind limbs in CIA mice after treatment with different formulations. (**b**) H&E and safranin-O (SO-FG) staining images of histological sections in joints, and the corresponding scores. Scale bar: 100 μm. Black arrows indicated bone destruction and loss of proteoglycan. (**c**) TNF-α and IL1-β immunohistochemical staining tissue sections of the knee joints of different groups. Scale bar: 100 μm. (**d**) Inhibition of HIF-1α (red) expression in the knee joint after different treatments. Scale bars: 100 μm. (**e-f**) Immunofluorescence images of M1 (CD86, red) and M2 (CD206, green) macrophage markers in the synovium after different treatments. Scale bars: 100 μm. Data were expressed as the mean ± SEM (*n* = 6, *p < 0.05, **p < 0.01, ***p < 0.001)

To further investigate the therapeutic effect, cartilage destruction and synovial inflammation of joint tissues [46] were evaluated by implementing Micro-CT, H&E staining, safranin-O staining and immunostaining, respectively. As depicted in Fig. 6a and S15, Micro-CT measurements showed that the bone erosion in the BSA@NPs-MTX MNs mice was minimal, with a smooth bone surface and adequate bone mass, similar to that in the normal group. To verify the pathological status of RA at the end of treatment, histological analysis was performed on mouse knee joint sections. As shown in Fig. 6b, H&E staining revealed that the inflamed joints in the model group exhibited obvious cartilage destruction, extensive inflammatory cell infiltration, and neovascularisation. In contrast, the joint cavity surface in the BSA@NPs-MTX MNs group showed clear boundaries and the most minimal synovial hyperplasia, similar to that in the normal group. In addition, safranin-O staining showed that proteoglycans in the model group were significantly lost, indicating severe cartilage damage. By contrast, the BSA@NPs-MTX MNs group exhibited better cartilage preservation. The quantification of cartilage structure was conducted utilizing Mankin’s scoring system [47].

Pro-inflammatory cytokines, such as TNF-α and IL-1β, were potential therapeutic targets for RA [48]. Therefore, the anti-inflammatory effects of BSA@NPs-MTX MNs were further evaluated by histological examination of pro-inflammatory cytokine expression. As shown in Fig. 6c, comparing to the normal group, the expression of TNF-α in the model group was significantly increased, indicating that TNF-α promoted the occurrence and development of RA. After treatment, the BSA@NPs-MTX MNs group showed a significant decrease of TNF-α expression than those of the MTX MNs and BSA@NPs MNs groups. The expression of the pro-inflammatory cytokine IL-1β was also reduced, consistent with the observed decrease in synovial inflammation and cartilage erosion. As shown in Figure S16, no significant pathological differences were observed in the main organs (heart, liver, spleen, lungs, and kidneys) in each group. This result verified that MTX MNs, BSA@NPs MNs, and BSA@NPs-MTX MNs had no obvious toxicity to the main organs, demonstrating good biocompatibility.

Furthermore, a basic analysis of the therapeutic mechanisms of the material was conducted, such as oxidative stress and macrophage transformation, among others. Immunofluorescence staining of HIF-1α in the knee joint showed that BSA@NPs-MTX MNs successfully reduced the expression of HIF-1α in knee tissue (Fig. 6d). Thus, BSA@NPs-MTX alleviated hypoxia around the RA synovial joint, subsequently shifting the macrophage phenotype from the pro-inflammatory M1 subtype to the anti-inflammatory M2 subtype. BSA@NPs-MTX, as a ROS scavenger, induced the transition of knee joint macrophages in RA from the M1 phenotype to the M2 phenotype. The levels of M1 macrophage-specific biomarkers, such as CD86, increased after RA induction; however, treatment with BSA@NPs-MTX significantly reduced these levels (Fig. 6e). As expected, RA also stimulated the expression of M2 markers owing to macrophage infiltration, and BSA@NPs-MTX further upregulated the anti-inflammatory M2 marker (CD206) (Fig. 6f, S17). Thus, BSA@NPs-MTX were demonstrated that can effectively suppress oxidative stress, transform M1-type macrophages into M2-type macrophages, greatly enhancing arthritis repair, and holding great prospects for applications in inflammatory diseases.

## Conclusion

In summary, we successfully prepared a programmable polymer MN system infused with BSA@NPs-MTX for efficient transdermal drug delivery in RA treatment. This study substantiated the remarkable efficacy of BSA@NPs-MTX MNs in eliminating a variety of reactive oxygen. MNs could penetrate the stratum corneum and systematically degrade within the skin tissues to release therapeutic agents, which further reinforced the potential of this innovative approach. Using RA mice as a model, this study validated that BSA@NPs-MTX MNs could eradicate ROS, augment oxygen levels, promote beneficial M2 macrophages, and diminish inflammatory M1 macrophages. These findings could markedly advance the current understanding and methodology of RA treatment, offering a pioneering strategy combining transdermal drug delivery and anti-inflammatory therapy as a promising new direction for future RA management and treatment.

### Electronic supplementary material

Below is the link to the electronic supplementary material.

Supplementary Material 1

## Data Availability

All data generated or analyzed during this study are available from the corresponding author on reasonable request.
