# Supplementary material for: Microneedles loaded with cerium-manganese oxide nanoparticles for targeting macrophages in the treatment of rheumatoid arthritis
Source: J Nanobiotechnology. 2024 Mar 11;22:103. doi: 10.1186/s12951-024-02374-y (PMC10926598; doi:10.1186/s12951-024-02374-y)
Supplement: Supplementary file 1 — Supplementary Material 1 [file 12951_2024_2374_MOESM1_ESM.docx]

**Supplementary material**

**Microneedles loaded with cerium-manganese oxide nanoparticles for targeting macrophages in the treatment of rheumatoid arthritis**

Tian Xia^1,2^†, Yuting Zhu^2,4^†, Kaiqiang Li^1^, Ke Hao^1^, Yingqian Chai^2,4^, Hongyi Jiang^2,4^, Chao Lou^2,4^, Jiachen Yu^2,4^, Wei Yang^5^, Jilong Wang^2,4^*, Junjie Deng^2,4^*, Zhen Wang^1,3^*

†Tian Xia and Yuting Zhu have contributed equally to this work.

*Correspondence: [wangjilong@ucas.ac.cn](mailto:wangjilong@ucas.ac.cn); j.deng@ucas.ac.cn;

[wangzhen@hmc.edu.cn](mailto:wangzhen@hmc.edu.cn)

^1^Laboratory Medicine Center, Allergy Center, Department of Transfusion Medicine, Zhejiang Provincial People's Hospital (Affiliated People's Hospital), Hangzhou Medical College, Hangzhou, Zhejiang310014, China.

^2^Joint Centre of Translational Medicine, Wenzhou Institute, University of Chinese Academy of Sciences, Wenzhou, Zhejiang 325000, China.

^3^Laboratory Medicine Center, Department of Transfusion Medicine, Tiantai People's Hospital of Zhejiang Province (Tiantai Branch of Zhejiang Provincial People's Hospital), Hangzhou Medical College, Taizhou, Zhejiang 317200, China.

^4^Zhejiang Engineering Research Center for Tissue Repair Materials, Wenzhou Institute, University of Chinese Academy of Sciences, Wenzhou, Zhejiang 325000, China.

^5^Department of Biophysics, and Department of Neurology of the Fourth Affiliated Hospital, Zhejiang University School of Medicine, Hangzhou, 310000, China.

Full list of author information is available at the end of the article

**Figure S1.** Quantitative fluorescence intensity analysis of intracellular ROS in LPS-activated RAW264.7 cells. Data were expressed as the mean ± SEM (n = 3, *p < 0.05, **p < 0.01, ***p < 0.001).


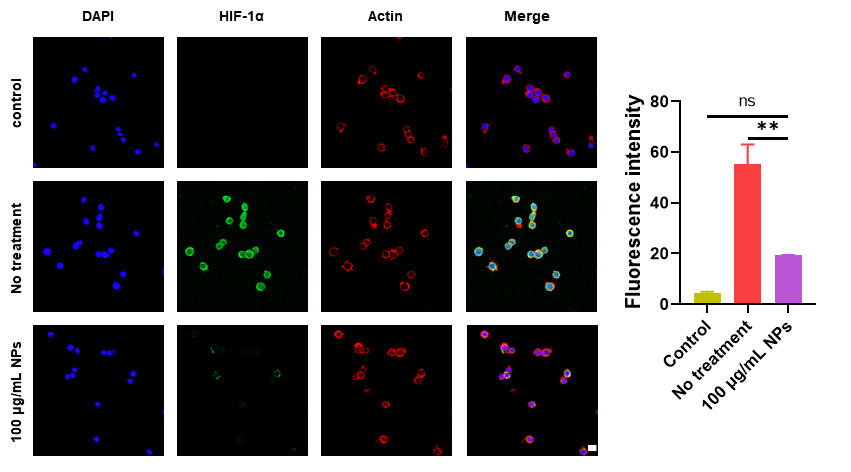


**Figure S2.** HIF-1α (green) staining of RAW264.7 cells incubated for 4 h under hypoxic and inflammatory (+ LPS) situations and subsequently treated with NPs for 4 h. Nuclei were stained with DAPI (blue) and F-actin (red) were used for visualization of cell structure. Scale bars: 10 µm. Data were expressed as the mean ± SEM (n = 3, *p < 0.05, **p < 0.01, ***p < 0.001, ns = no significance).


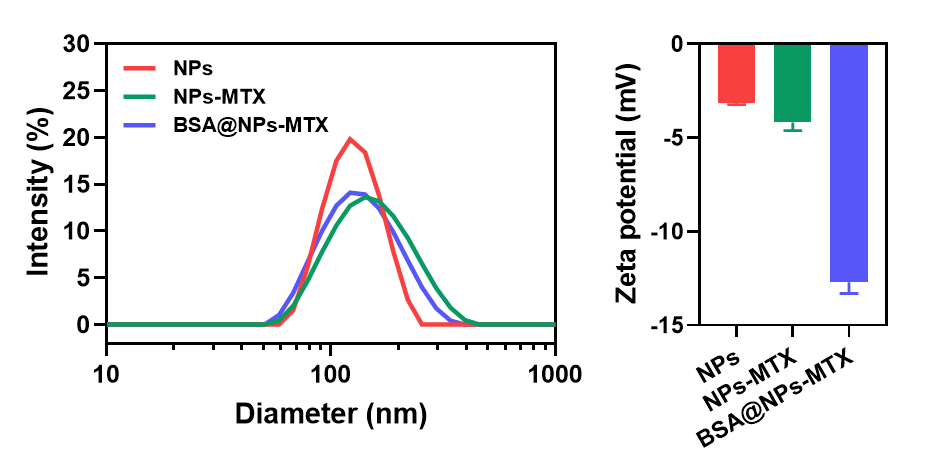


**Figure S3.** Particle size and zeta potential of the NPs, NPs-MTX, and BSA@NPs-MTX. Data were expressed as the mean ± SEM (n = 3).

**Figure S4.** Size stability of BSA@NPs-MTX in H_2_O, NaCl, and PBS buffer containing 10% FBS for 7 days. Data were expressed as the mean ± SEM (n = 3).

**Figure S5.** Ultraviolet-visible spectra of BSA@NPs-MTX.

**Figure S6.** FTIR spectra of MTX(a) and BSA@NPs-MTX(b).

**Figure S7.** Release curve of MTX from BSA@NPs-MTX. Data were expressed as the mean ± SEM (n = 3).

**Figure S8.** Cytotoxicity viability of RAW264.7 cells treated with various concentrations of BSA@NPs-MTX. Data were expressed as the mean ± SEM (n = 3).


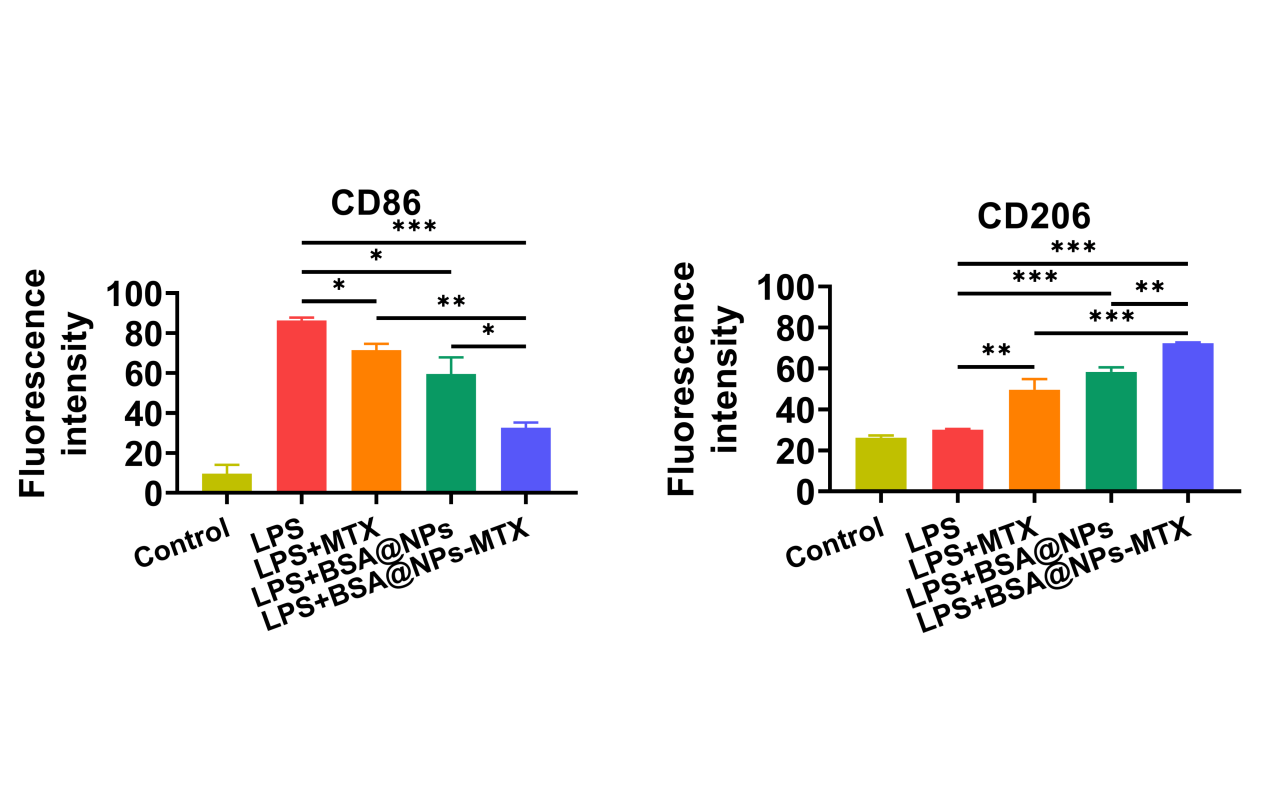


**Figure S9.** Quantitative analysis of CD86 and CD206 fluorescence intensity. Data were expressed as the mean ± SEM (n = 3, *p < 0.05, **p < 0.01, ***p < 0.001).


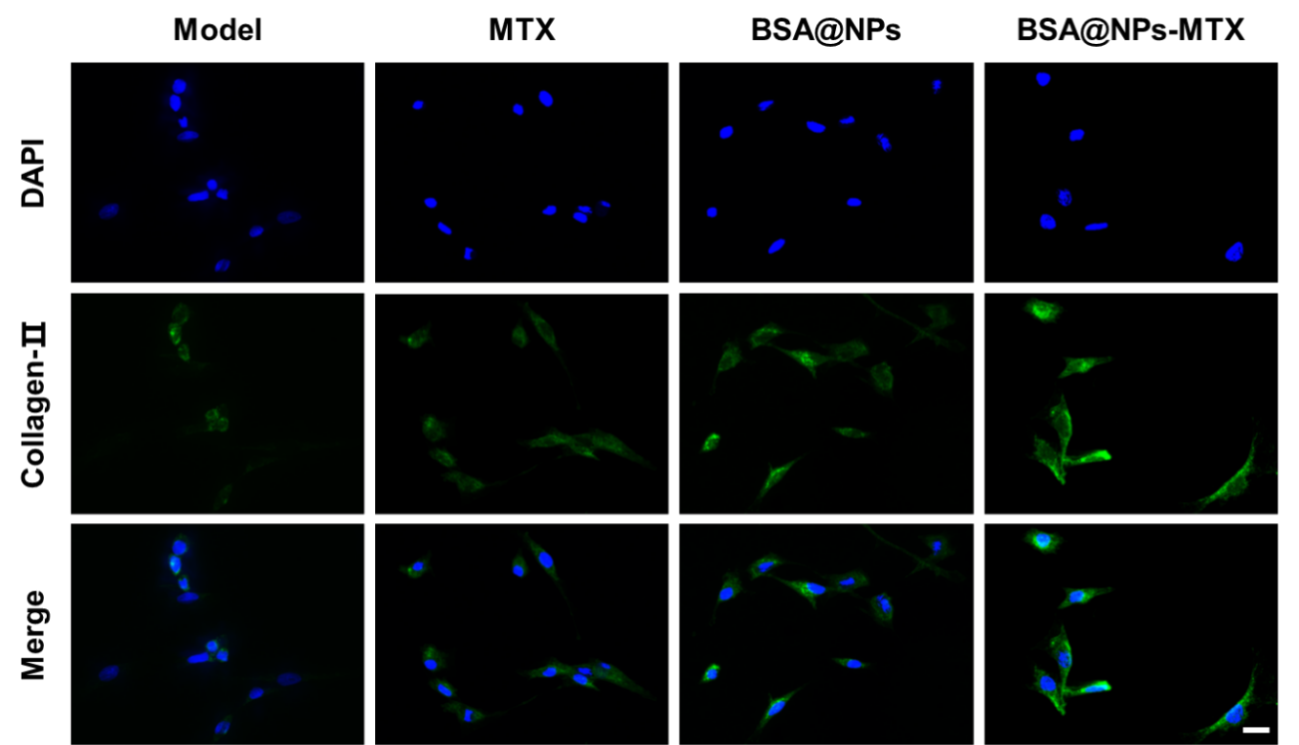


**Figure S10.** Expression level of collagen II was tested by immunofluorescent staining. Scale bars: 20 µm.


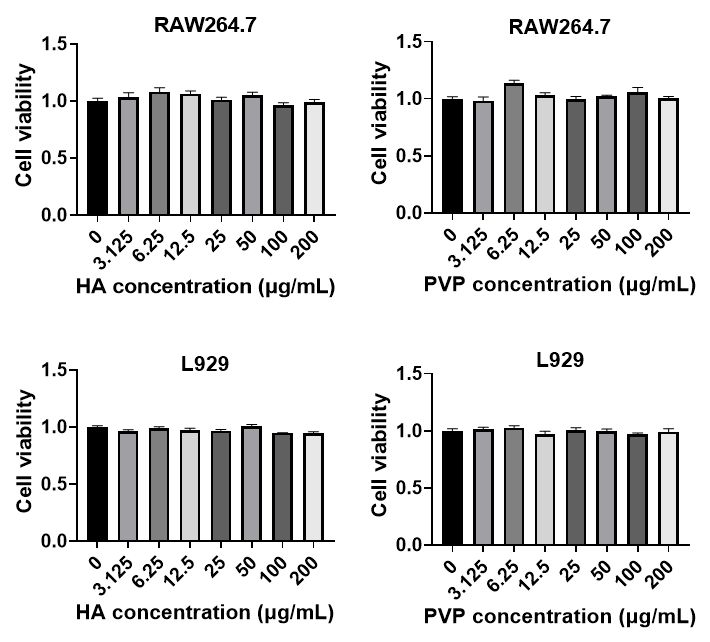


**Figure S11.** Cytotoxicity assay of MNs and materials which constituted MNs solution at different concentrations for 24 h. Data were expressed as the mean ± SEM (n = 3).


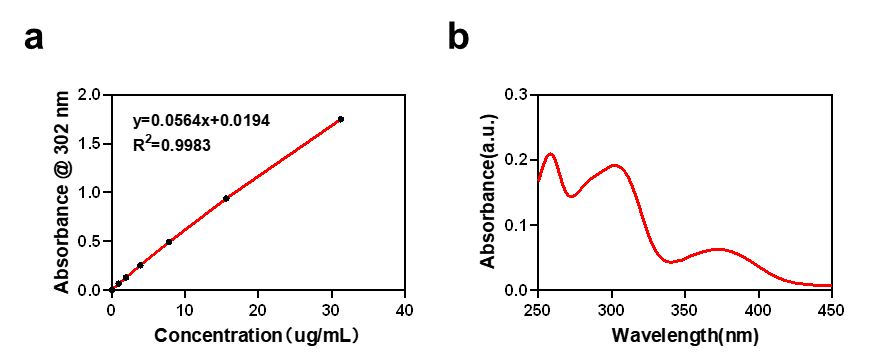


**Figure S12.** (a) Absorbance of MTX at 302 nm in a concentration-dependent manner. (b) The UV-vis absorption of MTX in the MNs.


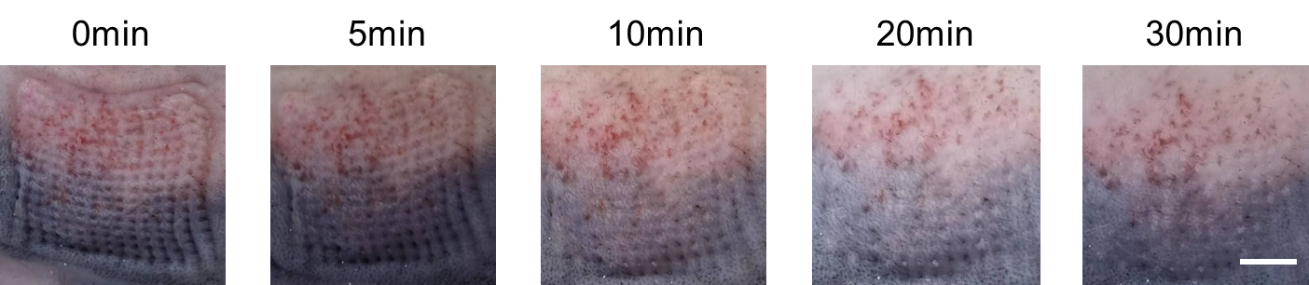


**Figure S13**. Skin recovery images and safety evaluation after MNs treatment. Scale bar: 2 mm.


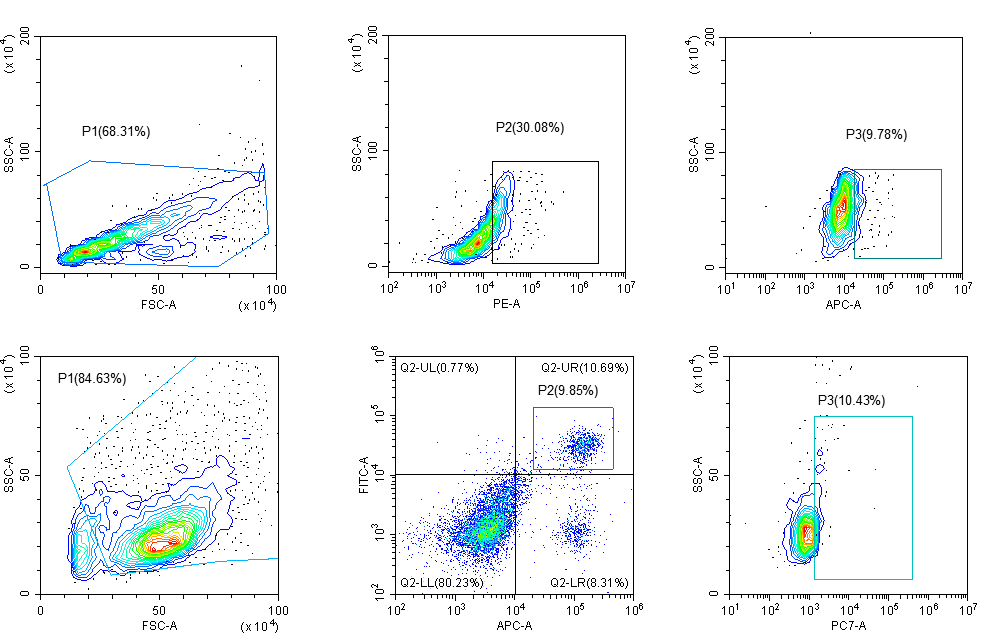


**Figure S14.** Representative flow cytometry plots of macrophages isolated from the liver and T lymphocytes isolated from the spleen were obtained after different treatments in mice.

**Normal**

**Model**

**MTX MNs**

**BSA@NPs MNs**

**BSA@NPs-MTX MNs**


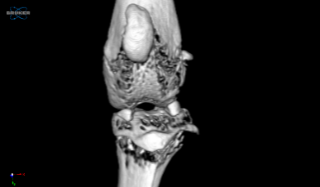

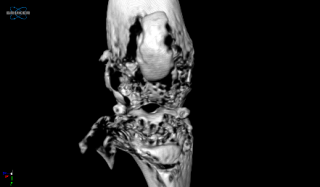

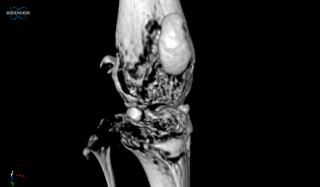

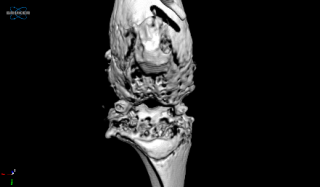

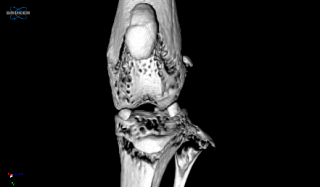


**Figure S15.** Representative Micro-CT images of the knee joint**.**


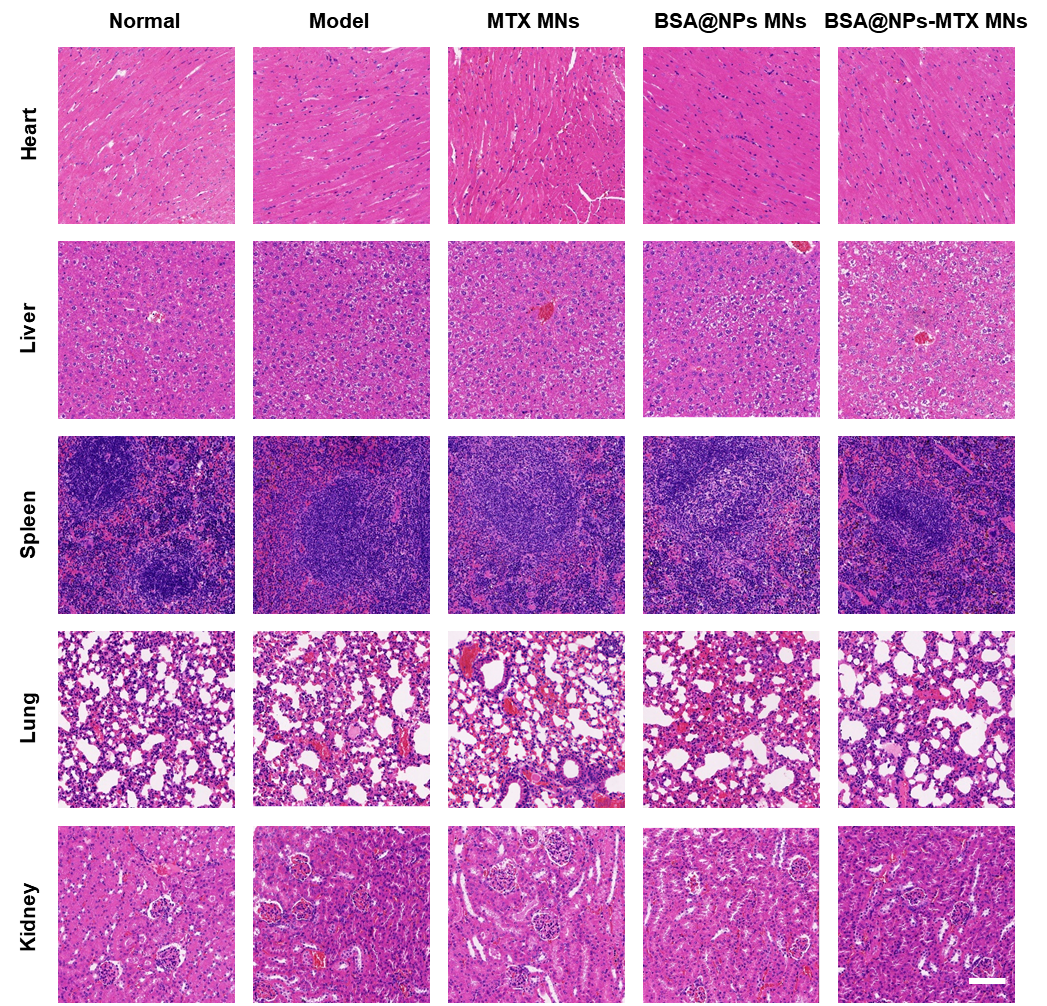


**Figure S16.** H&E staining of major organs of collected from different groups of mice. Scale bars: 100 µm.


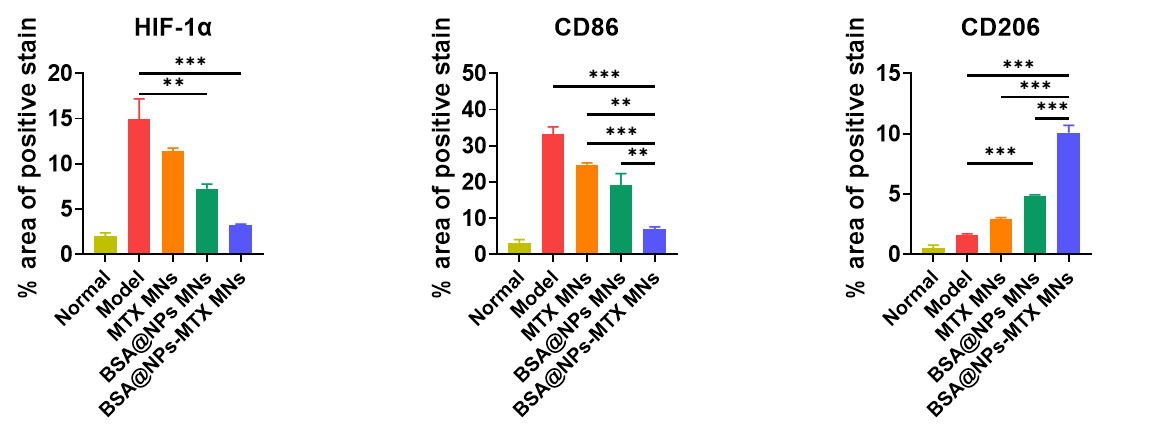


**Figure S17.** The corresponding immunofluorescence intensity quantification of HIF-1ɑ, CD86 and CD206. Data were expressed as the mean ± SEM (n = 6, *p < 0.05, **p < 0.01, ***p < 0.001).

**Table S1: Primers used in qRT-PCR**

| Corresponding gene name Primer sequences | |
| --- | --- |
| GAPDH | F: 5'-TGTGGATGGCCCCTCTGGAA-3'  R: 5'-TGACCTTGCCCACAGCCTTG-3' |
| iNOS  IL-6  TNF-α  IL-10  ARG-1  TGF-β | F: 5'-GAGACGCACAGGCAGAGG-3'  R: 5'-CAGGCACACGCAATGATGG-3'  F: 5'-CTGGAGCCCACCAAGAACGA-3'  R: 5'-GCCTCCGACTTGTGAAGTGGT-3'  F: 5'-AGGGTCTGGGCCATAGAACT-3'  R: 5'-CCACCACGCTCTTCTGTCTAC-3'  F: 5'-CCAGTACAGCCGGGAAGACA-3'  R: 5'-GAAGGCAGTCCGCAGCTCTA-3'  F: 5'-TTGGGTGGATGCTCACACTG-3'  R: 5'-GTACACGATGTCTTTGGCAGA-3'  F: 5'-GCCACTGCCCATCGTCTACT-3'  R: 5'-CACTTGCAGGAGCGCACAAT-3' |
